# Improving Diagnostic Accuracy for Distinguishing Buckle Fractures From Other Distal Radius Fractures in Children

**DOI:** 10.1097/pq9.0000000000000547

**Published:** 2022-03-30

**Authors:** Lynne Ruess, Margarita Chmil, Satbir Singh, Julie B. Samora

**Affiliations:** * Department of Radiology, Nationwide Children’s Hospital, Columbus, Ohio; † Department of Radiology, The Ohio State University College of Medicine, Columbus, Ohio; ‡ Department of Orthopedic Surgery, Nationwide Children’s Hospital, Columbus, Ohio; § Department of Orthopedic Surgery, The Ohio State University College of Medicine, Columbus, Ohio.

## Abstract

**Introduction:**

Accurately distinguishing between stable and unstable isolated distal radius fractures (DRF) in children allows for appropriate fracture-specific treatment. Although fractures with cortical disruption, displacement, or angulation are unstable, distinguishing stable buckle fractures (BF) from more subtle potentially unstable DRF is challenging. Our quality improvement project aimed to improve radiology reporting accuracy for these subtle fractures from 23% to 90% in a large tertiary pediatric hospital.

**Methods:**

Exams with a reported isolated distal radius fracture during baseline (January–March 2016) and intervention (April 2016–June 2019) were reviewed for accuracy. We introduced 3 types of interventions: radiologist education (self-directed learning modules and individual feedback), a new standardized report template, and a measurement tool (“The 1 cm Rule”). In addition, a statistical process control chart tracked accuracy data to study process changes over time.

**Results:**

During the baseline and intervention period, 22 and 480 radiographs, respectively, had either a stable BF or a potentially unstable isolated DRF. Each intervention type created a centerline shift. Overall, reporting accuracy increased from 23% to 90%. Most reports (95%, 639/676) used the template and standard terminology for reporting DRF.

**Conclusions:**

Radiology reporting diagnostic accuracy for distinguishing between stable BF and potentially unstable DRF in children increased to 90% through education, standardized reporting, and a measurement tool to enhance radiologist performance. Our institution plans to expand fracture-specific treatment practices with improved radiology reporting accuracy, including bracing and home management of stable BF diagnosed during an acute care visit.

## INTRODUCTION

Forearm fractures are common injuries encountered in pediatric emergency departments and urgent care centers. The standard of care has been to immobilize all pediatric forearm fractures in a splint or cast with orthopedic follow up. More recently, managing these injuries has become fracture specific. For example, treatment of the stable distal radius buckle fracture (BF), which manifests as a subtle bone deformity without cortical disruption, is trending toward home management with a removable wrist brace. In contrast, other clearly unstable or potentially unstable distal radius fractures (DRF) still require a splint or cast immobilization and orthopedic follow up.^1–7^ This change to bracing for BF is desirable for children and families who prefer convenient home treatment without required follow-up appointments.^3,4,8^

**Fig. 1.**
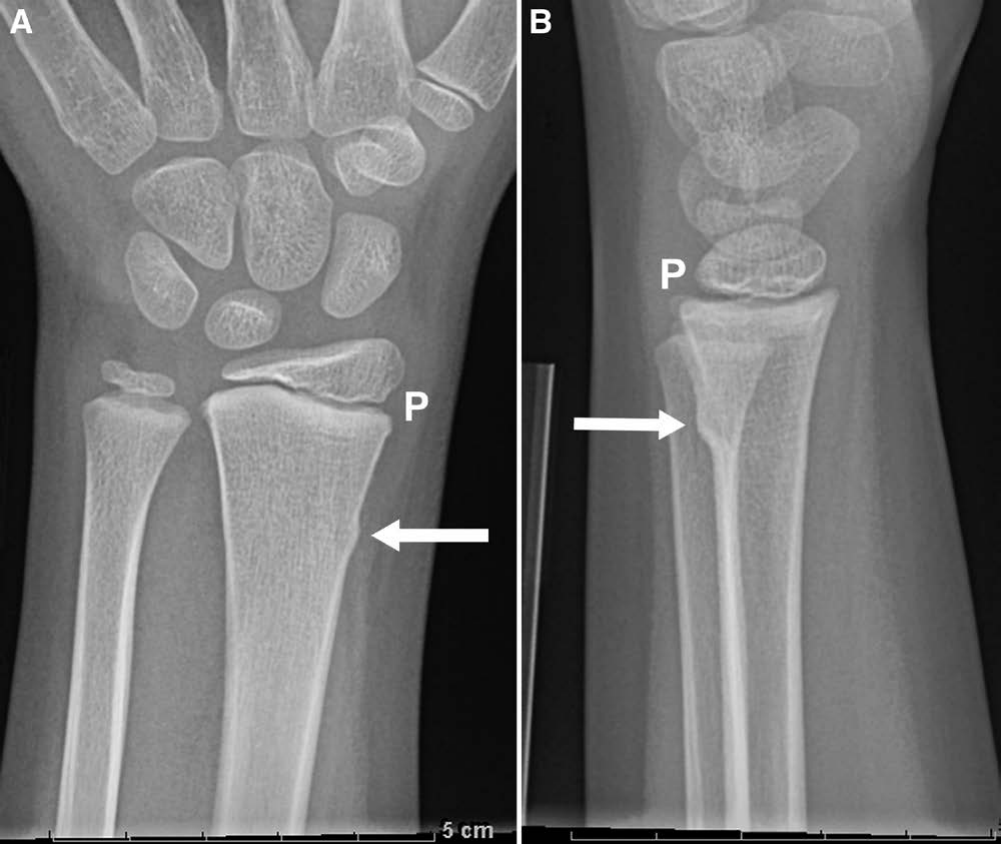
Eight-year-old boy with BF. Posteroanterior (A) and lateral (B) views of the wrist show a cortical buckle deformity with normal bone between the fracture (arrow) and the distal radial physis (P).

**Fig. 2.**
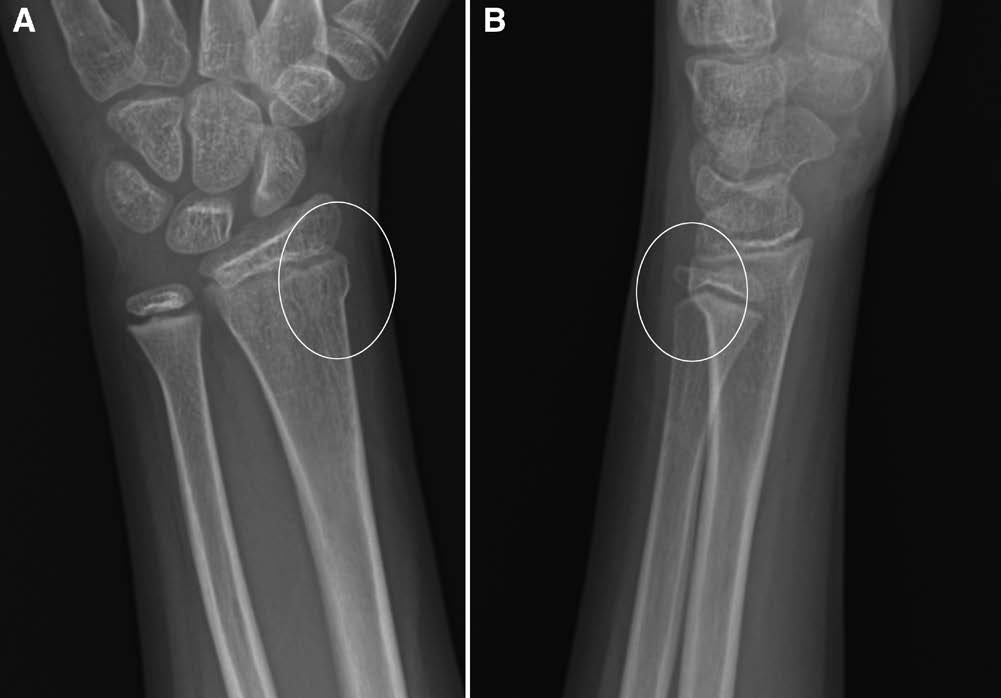
Thirteen-year-old girl with potentially unstable DRF. Posteroanterior (A) and lateral (B) views of the wrist show distal radius cortical deformity adjacent to the physis (circle). The cortex is intact.

Accurately distinguishing between BF and DRF at the time of diagnosis is imperative for appropriate treatment. At our institution, wrist or forearm radiographs are typically ordered by acute care triage professionals, with the final radiology report available to physicians at the time of management decision making. At baseline, most of our radiologists reported descriptive statements about DRF but did not provide a specific fracture diagnosis. Following a request from our orthopedic colleagues to make a specific diagnosis, radiology reporting accuracy varied. Accuracy was high for clearly unstable DRF, including displaced fractures, angulated fractures, or cortical disruption. However, the diagnosis was more challenging, and accuracy was low when radiologists relied on subtle morphologic differences to distinguish between BF (Fig. 1) and potentially unstable DRF types without cortical disruption or a clear fracture lucency extending to the physis (Fig. 2). This diagnostic imprecision prompted a measurement guideline called “The 1 cm Rule” to aid diagnosis.^9^

### Specific Aim

Using quality improvement (QI) methodology, our project aimed to improve the radiology report diagnostic accuracy for stable BF and subtle potentially unstable DRF in children 3–16 years, from 23% to 90% in 1 year.

## METHODS

### Context

Our institution consists of a large urban pediatric tertiary care hospital with 2 freestanding emergency departments and 8 urgent care centers in surrounding communities. The orthopedic case volume is high, with 22 pediatric radiologists providing 24/7 timely interpretations and reports for all orthopedic exams. This QI work was exempt from IRB review per institutional policy. Article preparation followed the Standards for Quality Improvement Reporting Excellence (SQUIRE) 2.0 guidelines.^10^

### Clinical Care

Final radiology reports are typically available to the acute care provider before discharge. During the baseline period, all DRF were casted. The preferred treatment for a stable BF was a splint or brace during the intervention period, although all clearly unstable or potentially unstable DRF were casted. During the baseline and intervention periods, all children diagnosed with an isolated distal radius fracture in an emergency department or urgent care center had a follow-up appointment in the orthopedic clinic within 1 week.

### Data Collection

Patient radiographic exams were identified via electronic medical records (EPIC Systems, Corp., Verona, WI). For baseline data, we reviewed initial injury forearm and wrist radiographs with a reported radius fracture in children (3−16 years) evaluated in one of our emergency departments or urgent care centers between January 1, 2016, and March 30, 2016. Subsequent data searches provided exams from April 1, 2016, through June 30, 2019. We excluded exams with angulated or displaced distal third radius fractures, middle third, or proximal radius fractures or exams with >1 fracture, including ulna fractures. We also excluded exams initially interpreted by the first author (L.R.).

**Fig. 3.**
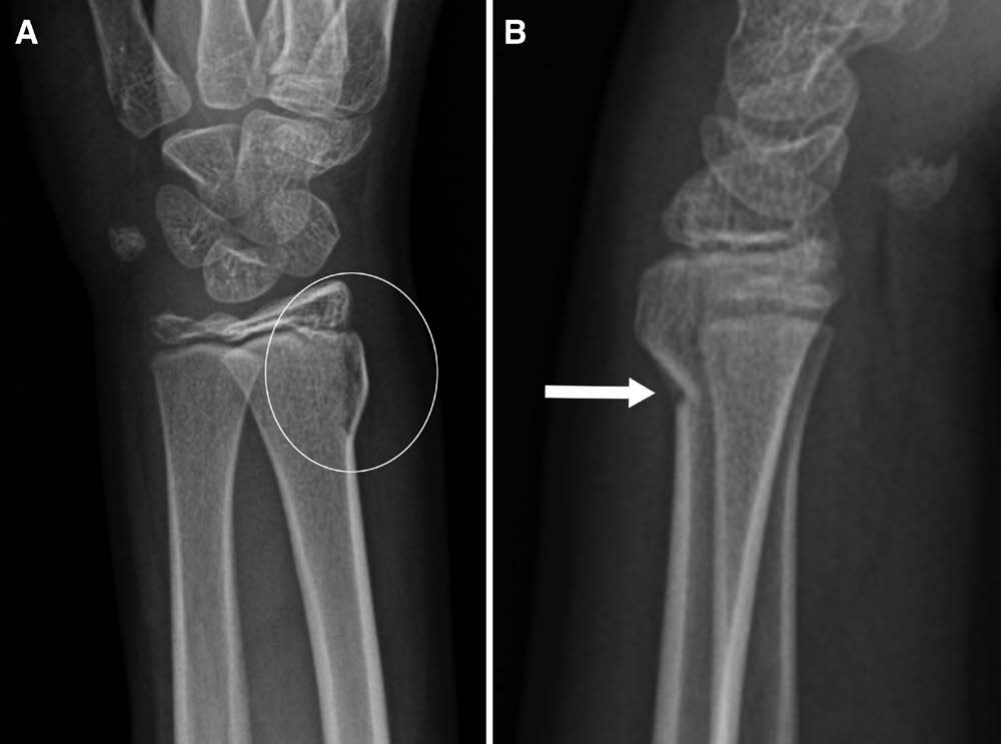
Ten-year-old boy with unstable DRF. Posteroanterior (A) and lateral (B) views of the wrist show both cortical disruption (B, arrow) and fracture lucency extending to the distal radial physis (A, circle).

Consensus established the final diagnosis between author L.R. (pediatric radiologist, 25 years experience) and author J.B.S. (orthopedic hand surgeon, 8 years experience) and the treating orthopedic provider in the follow-up clinic. We defined a stable BF as an incomplete fracture with no cortical disruption and normal bone between the fracture and the physis (Fig. 1A,B). We classified all other isolated distal radius non-BF as potentially unstable DRFs (Fig. 2A,B), defined by cortical disruption, or a fracture lucency extending to the physis (Fig. 3A,B). For a process measure, we reviewed radiology reports from January 1, 2017, to June 30, 2019, to evaluate compliance using the suggested “Final Impression” terminology when reporting any distal radius fracture.

### Interventions

**Fig. 4.**
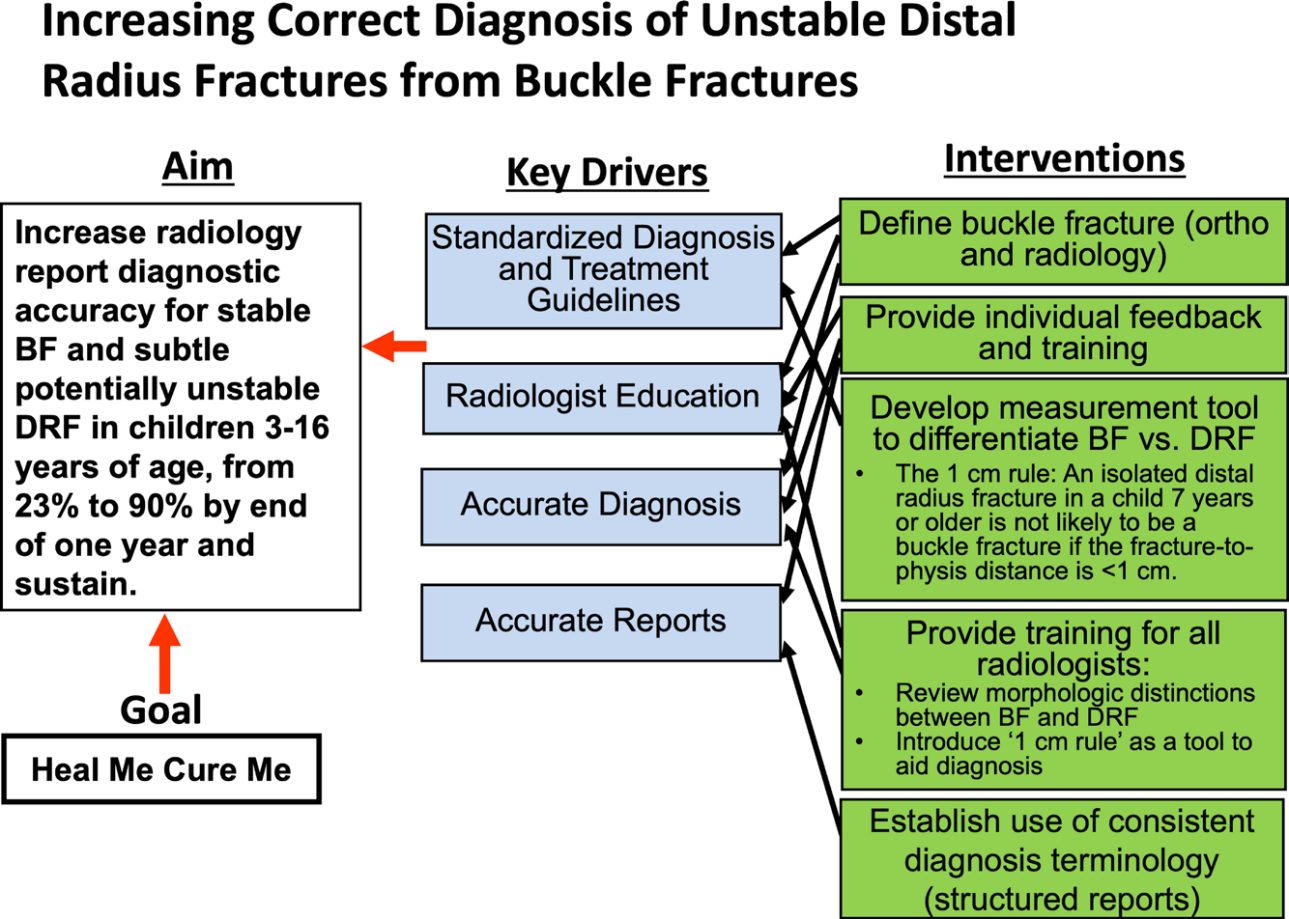
Key driver diagram outlines the aim, key drivers, and interventions for increasing radiology report diagnostic accuracy for stable BF and subtle potentially unstable DRF in children 3–16 y.

The interventions are outlined in the key driver diagram (Fig. 4). In April 2016, the orthopedic service asked the radiologists to use “buckle fracture” when appropriate. Education for radiologists began in July 2016 with a formal online training module using the REDCap electronic data capture tool hosted by our institution.^11^ The first intervention module reviewed fracture nomenclature and the morphologic criteria for BF diagnosis. Radiologists also learned of the institutional practice changes regarding treatment and follow up for BF versus other DRF. The module included a 12 question pretest and a 12 question posttest. The REDCap reminder feature ensured 100% participation. New radiologists who joined the group also completed this training during the intervention period. In September 2016, all radiologists completed a follow-up 26 case-based quiz with immediate feedback, including an explanation for each answer using the REDCap tool with 100% participation.^11^

In December 2016, we created a standardized reporting template to improve diagnosis communication with the emergency department and urgent care providers. The new forearm and wrist template provided a menu of consistent “Final Impression” terminology for the 3 most common forearm fractures: radius fracture (nonbuckle), buckle fracture-distal radius, and both bone forearm fractures.

**Fig. 5.**
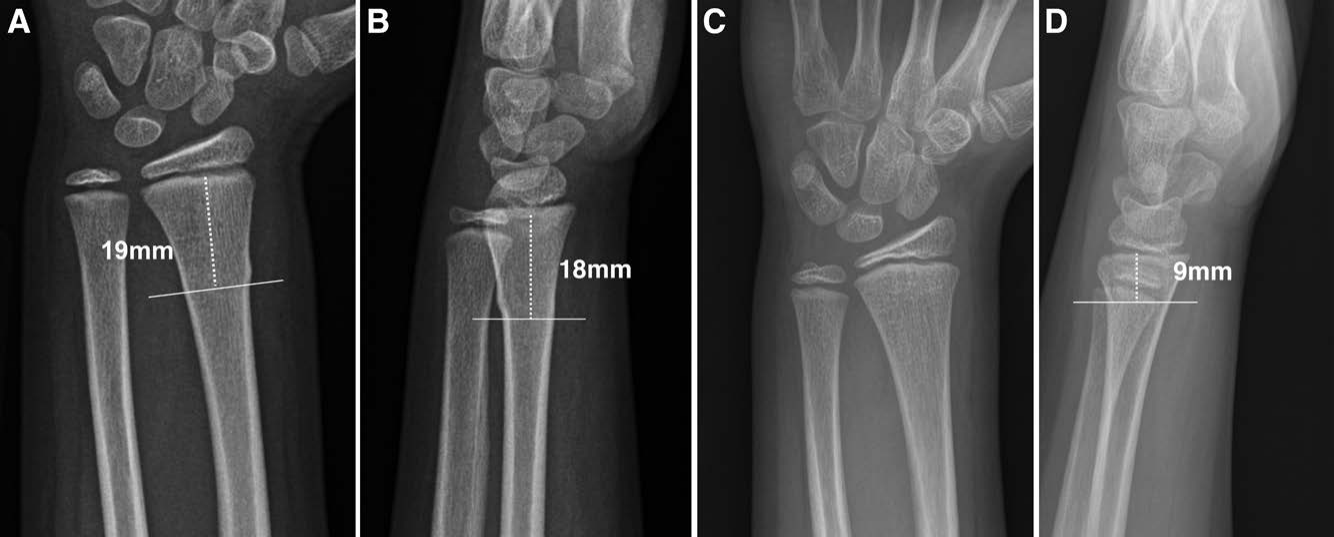
Illustration of fracture-to-physis distance measurement. (A, B) Eight-year-old girl with BF. The distance (dashed line) from a transverse line at the most proximal cortical deformity to the mid physis measures 19 mm on the posteroanterior (A) view and 18 mm on the lateral (B) view. (C, D) Ten-year-old girl with potentially unstable DRF. The fracture-to-physis distance is difficult to visualize and measure on the posteroanterior (C) view and is <1 cm (dashed line) on the lateral (D) view. “The 1 cm Rule” discourages the diagnosis as a BF if the fracture-to-physis distance is <1 cm.^9^

After 1 year, when accuracy had not yet reached the goal, we developed a measurement guideline as a tool to aid in diagnosis.^9^ In April 2018, based on measuring the fracture-physis-distance in over 200 children, we introduced “The 1 cm Rule” to our radiologists. This rule states an isolated distal radius fracture in a child is not likely to be a BF and should instead be considered a potentially unstable DRF if the fracture-to-physis distance is <1 cm,^9^ particularly in children 7 years or older. That month, all radiologists completed a third updated online training module, which included measurement instructions (Fig. 5A–D), and reference posters were created and displayed in the radiologists’ work areas.

In January 2019, all radiology department reporting templates underwent further revision, including forearm and wrist exams. Specifically, rather than text descriptions of fractures in the body of the report, a subtemplate required fill-in for angulation and displacement details for each fracture. However, the previously edited “Final Impression” menu options for the common forearm fractures did not change. That month, the first author L.R. presented the revised fracture reporting template, along with a review of BF versus DRF fracture diagnosis criteria during a radiology department conference; 10/22 (45%) attended live, and the remaining were able to access it from the department electronic shared folder.

Throughout the intervention period, radiologists received individual feedback via email for incorrect diagnoses identified by orthopedic providers or during periodic peer review. They also received personal feedback for discrepancies noted at batch review (January–April 2018, November 2018, and June 2019).

### Analysis

The number of correct diagnoses was determined by comparing the radiology report diagnosis and the final diagnosis. We calculated accuracy using the number of accurate diagnoses as the numerator and the total number of DRF without cortical disruption or fracture lucency extending to the physis as the denominator. A radiology quality coordinator (MC) loaded accuracy data into a proprietary statistical process control charting template, which allowed the team to study process changes over time.^12^ Annotated charts indicated the timing of interventions.

We utilized the American Society for Quality (ASQ) criteria for adjusting the centerline and control limits for the statistical process control chart. In addition, we reviewed the process stages for variations.^13^ Diagnostic accuracy was determined in monthly intervals. In addition to the monthly QI team reports and discussions, the data were presented to the Director of Quality Improvement and the Chief Medical Officer at our institution for periodic review.

## RESULTS

### Study Population

We reviewed 54 radiographs from the baseline period and 943 from the intervention period. After eliminating the obvious unstable DRF, 22 radiographs in the baseline period and 480 in the intervention period resulted. Patient age (3−16 years) and distribution were similar in the baseline and intervention groups with a mean of 9.6 ± 3.6 years and 9.6 ± 3.2 years, respectively (*P* = 0.893).

### Radiology Reporting Accuracy

**Fig. 6.**
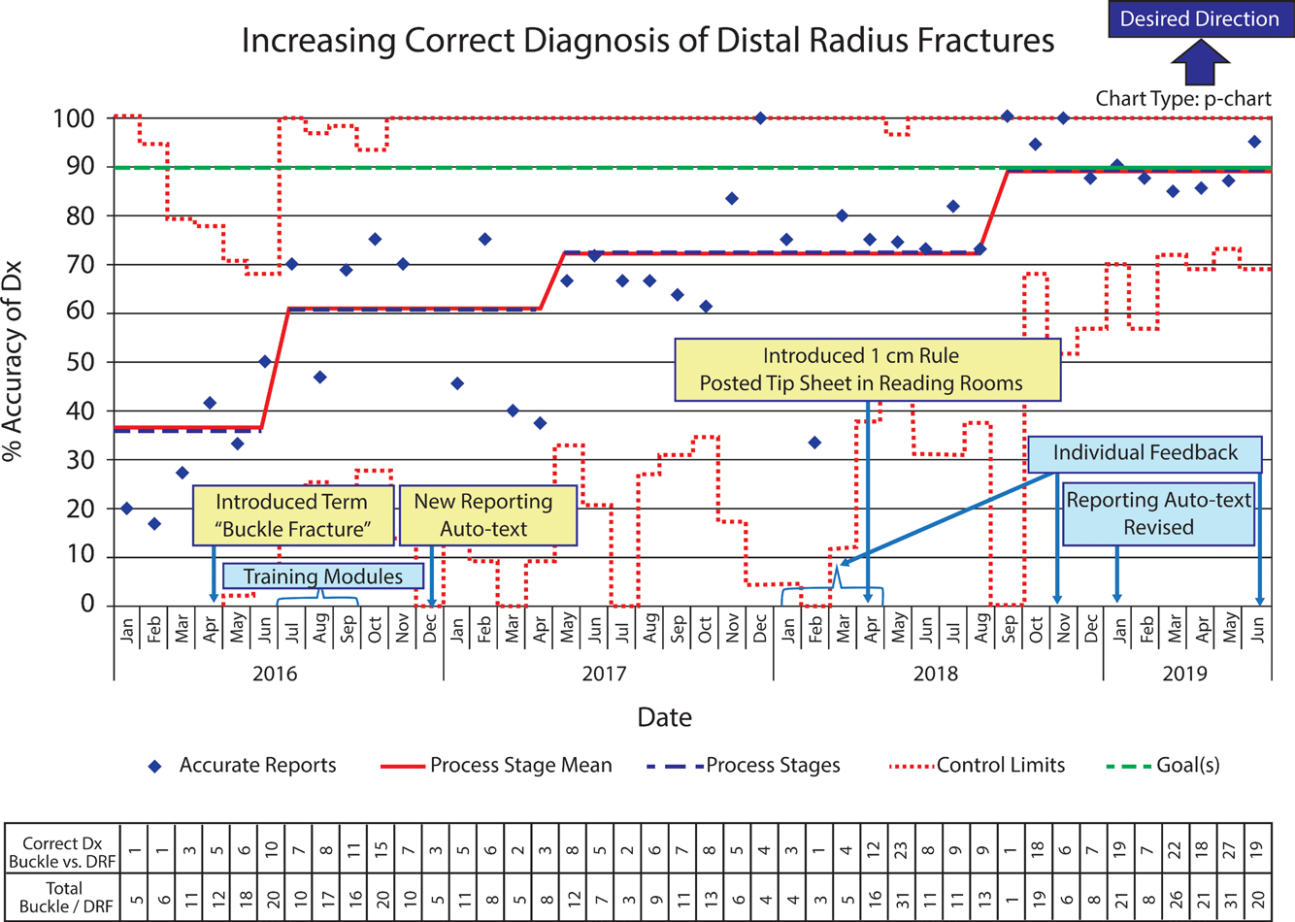
P-chart shows the diagnostic accuracy change for stable buckle fractures and potentially unstable isolated distal radius fractures in children over time. Interventions are indicated in blue and yellow text boxes.

Diagnostic reporting accuracy was 100% for clearly unstable DRF (32/32) and only 23% (5/22) for BF versus potentially unstable DRF during the 3-month baseline period. Diagnostic accuracy remained at 100% for clearly unstable fractures, including displaced fractures, angulated fractures, or those with cortical disruption (463/463), whereas reporting accuracy for stable BF and potentially unstable DRF increased with 3 process shifts during the intervention period (Fig. 6). With the start of the first radiologist training module in July 2016, a significant improvement based on ASQ criteria was evident.^13^ With the associated control limits, the centerline adjusted from 36% to 61% (κ= –0.247 to 0.176). After a new reporting template with standardized terminology became available in December 2016, the centerline shifted to 72% (κ = 0.447). Finally, after introducing “The 1 cm Rule” in April 2018, the centerline shifted a third time to 90% (κ = 0.788). Average diagnostic accuracy for the last 10 months was 92% (22/24) for children (3−6 years) and 89% (122/137) for children (7−16 years). There were 17 (10%) false-positive diagnoses for BF during this time.

### Process Measure

After introducing the radiology reporting template for the “Final Impression” section, most (95%, 639/676) reports included the template or standard terminology for reporting an isolated distal radius fracture, either a BF or a nonbuckle type DRF.

## DISCUSSION

This project was part of a joint initiative with orthopedic surgery to provide fracture-specific care for stable BF and potentially unstable DRF in children evaluated and treated in a large tertiary hospital system. Although diagnostic reporting accuracy was very high at baseline for clearly unstable DRF, we aimed to improve radiology report diagnostic accuracy to distinguish between stable BF and subtle, potentially unstable DRF types using QI methodology. As a result, we increased diagnostic accuracy for these fractures from 23% to 90%.

Our process started with required education modules for all radiologists and, although not explicitly measured, individual feedback was ongoing throughout the intervention period. The second intervention replaced descriptive reporting with standardized reporting requiring clear and concise terminology within the radiology report’s “Final Impression” section. Because our radiologists were familiar with the format of standardized templates, this intervention was readily accepted. Our final intervention introduced the “1 cm Rule” after testing an observation that the cortical deformity of a stable BF tended to occur at a greater distance from the physis than nonbuckle DRF.^9^ This rule discourages diagnosing a BF if the fracture-to-physis distance is <1 cm. After providing radiologists with this measurement tool, diagnostic accuracy improved to meet the 90% goal.

At our institution, fracture-specific treatment for DRF began with BF bracing during the intervention period. Children (3−16 years) diagnosed with an acute BF in the emergency department, or urgent care was fitted with a removable splint or brace and discharged with a scheduled orthopedic follow-up appointment to confirm appropriate diagnosis and treatment. With improved diagnostic accuracy at the time of presentation, fewer children with BF required a change in diagnosis and management at the follow-up visit. We did not include fractures in children under 3 years because even the smallest commercially available braces are generally too large for young children. Also, we excluded fractures in older teens (older than 16 years) as BF does not typically occur in skeletally mature individuals.

There were 17 potentially unstable DRF misdiagnosed and initially managed as stable BF during the intervention period. These were recognized within 1 week of diagnosis (during either radiology peer review or more often during the scheduled follow-up appointment in the orthopedic clinic). At that time, the fracture was casted with routine follow up for a potentially unstable fracture. There were no complications in this group.

After improving radiology reporting accuracy through this project’s interventions, follow-up instructions for acute BF versus other DRF are now fracture specific. Children diagnosed with an acute BF in one of our emergency departments or urgent care centers will be discharged with a removable brace and instructions for home management. The written discharge instructions include a list of indications to return for assessment. However, patients with a stable BF will no longer require routine formal orthopedic follow up. This practice eliminates potential lost work and school time and is desirable for children and families.^3,4,8^ We plan to review the radiographs and reports of patients placed on this treatment pathway to ensure optimal patient care and continued reporting accuracy.

### Limitations

One limitation of this project is that the medical records were searched in batches, each with slightly different search parameters due to information system-search software changes. The 3 centerline shifts occurred within the period of search batches and not between them, suggesting that batched record searching did not contribute to improved reporting accuracy. The search parameter differences might account for variation in monthly volumes as a repeat search of 3 months in the midintervention period identified additional children with DRF. However, including these other exams did not affect the diagnostic accuracy (73% vs. 74%). Another limitation is that we did not utilize the traditional Plan-Do-Study-Act technique,^13^ with small incremental changes. Rather, we tracked our progress, changing the algorithm in stages with larger changes.

Finally, fracture-specific practice patterns may not be generalizable to other institutions. For example, in our hospital system, a staff pediatric radiologist reviews acute care musculoskeletal radiology exams within minutes; thus, the final radiology report guides treatment and discharge planning. Although this rapid radiology reporting may not be feasible in other acute care settings, training clinical providers to distinguish between these fracture types is possible. Also, our institution has a robust focus on QI with leadership support, a strong culture of patient safety, and abundant resources for QI work, which may not exist in other institutions.

## CONCLUDING SUMMARY

A quality initiative increased diagnostic accuracy for stable BF and potentially unstable DRF in children through education, standardized reporting, and a measurement tool to enhance radiologist performance. In addition, our institution plans to expand fracture-specific treatment practices with improved radiology reporting accuracy, including bracing and home management of stable BF diagnosed during an acute care visit.

## ACKNOWLEDGMENTS

The authors thank the teaching staff and other participants at our institution’s Quality Improvement Scientific Writing course, especially the numerous article reviews and suggestions provided by Anja Zann, MD.

## DISCLOSURE

The authors have no financial interest to declare in relation to the content of this article.
